# Catalogue of the type material of Phlebotominae (Diptera, Psychodidae) deposited in the Instituto Evandro Chagas, Brazil

**DOI:** 10.3897/zookeys.395.6417

**Published:** 2014-04-01

**Authors:** Thiago Vasconcelos dos Santos, Maria Sueli Barros Pinheiro, Andrey José de Andrade

**Affiliations:** 1 Laboratório de Leishmanioses Prof. Dr. Ralph Lainson, Instituto Evandro Chagas, Secretaria de Vigilância em Saúde, Ministério da Saúde. Rod. BR 316, Km 07 s/n. 67030-000, Levilândia, Ananindeua, PA, Brazil; 2 Departamento de Epidemiologia, Faculdade de Saúde Pública, Universidade de São Paulo, Avenida Doutor Arnaldo 715, 01246–904, São Paulo, SP, Brazil; 3 Laboratório de Parasitologia e Biologia de Vetores, Área de Patologia, Faculdade de Medicina, Universidade de Brasília, Campus Universitário Darcy Ribeiro, Asa Norte, 70910-900, Distrito Federal, Brazil

**Keywords:** Collection, sand flies, taxonomy, leishmaniasis

## Abstract

The available type material of Phlebotominae (Diptera, Psychodidae) deposited in the “Coleção de Flebotomíneos” of the Instituto Evandro Chagas (ColFleb IEC) is now presented in an annotated catalogue comprising a total of 121 type specimens belonging to 12 species as follow: *Nyssomyia richardwardi* (2 female paratypes), *Nyssomyia shawi* (9 male and 25 female paratypes), *Nyssomyia umbratilis* (female holotype and 1 female paratype), *Nyssomyia yuilli yuilli* (1 male and 1 female paratypes), *Pintomyia gruta* (1 male and 2 female paratypes), *Psychodopygus lainsoni* (2 male syntypes), *Psychodopygus leonidasdeanei* (male holotype, female “allotype” and 45 female paratypes), *Psychodopygus llanosmartinsi* (2 female paratypes), *Psychodopygus wellcomei* (1 male and 4 female “syntypes”), *Trichophoromyia readyi* (male holotype, female “allotype” and 1 male paratype), *Trichophoromyia adelsonsouzai* (male holotype, 13 male 5 female paratypes), and *Trichophoromyia brachipyga* (1 male paratype).

## Introduction

In the Americas, more than 450 species of the subfamily Phlebotominae Rondani, 1840 (Diptera: Psychodidae) have been described (Galati 2003) and 267 of these have so far been recorded in Brazil (Andrade et al. 2013). Phlebotomine sand flies play an important role in the transmission of virus, bacteria and protozoa, mainly *Leishmania* species. In Brazil six and eleven species have been incriminated or suspected, respectively, in the epidemiology of cutaneous leishmaniasis (Ready 2013). On the other hand and also in Brazil, the main vector of visceral leishmaniasis is the psychodid *Lutzomyia longipalpis* (Lutz & Neiva, 1912) while *Lutzomyia cruzi* (Mangabeira, 1942) and *Lutzomyia almerioi* Galati & Nunes, 1999 have been indicated as suspected vectors (Santos et al. 1998, Savani et al. 2009).

Biological collections are an important source of information on the composition, distribution and biological degree of biodiversity (Suarez and Tsutsui 2004). In addition, the types (the specimens described in the first published account of a new taxonomic group) deposited in these collections are the most valuable material for this information and their maintenance thus depends on financial support and curatorial care (NPS 2005).

In Brazil, there are six depositary institutions with type–species of sand flies. One of these is the “Coleção de Flebotomíneos” in the Instituto Evandro Chagas (IEC), known by the acronym “ColFleb IEC”, located in Ananindeua municipality, State of Pará. At present, this collection consists of approximately 140 *taxa* distributed in species and subspecies levels of Phlebotominae (Fraiha and Ryan 1986). Most of them were collected, mounted and deposited by previous researchers in the IEC during studies on the ecology and epidemiology of leishmaniasis in Amazonian Brazil (Lainson and Shaw 2005).

Some glass slide labels of non-type material revealed that the oldest specimens of this collection belong to the 1960’s (TVS, personal observation). Although a total of 55 valid species have been described by researchers directly related to the Instituto Evandro Chagas between 1945 and 2014 (Fraiha and Ryan 1986, Santos et al. 2014), some of these type specimens seem to have been lost, such as *Evandromyia carmelinoi* (Ryan, Fraiha, Lainson & Shaw, 1986). According to original description, some paratypes of *Bichromomyia olmeca nociva* (Young & Arias, 1982) and *Psychodopygus yucumensis* (Le Pont, Caillard, Tibayrenc & Desjeux, 1986) should have been deposited in the Instituto Evandro Chagas collection, but not one of these has been found.

This study presents an annotated catalog of the type material of the Phlebotominae deposited at the ColFleb IEC to help entomologists searching for types.

## Material and methods

Each type specimen was examined individually after checking the respective labels against original descriptions and additional bibliographical material. An active search for glass slides stored in boxes of nontype material was undertaken and the slides carefully analyzed to avoid excluding types which might have been mislaid.

After screening and comparison with original drawings, the type status of the specimens was confirmed and those identified were reorganized in horizontal slide cabinets with red labels for holotypes/“allotypes”; and green labels for paratypes/“syntypes”. The list presents basic information directly from the original labels (species name, date, locality and collector/determinator). Although some types were originally classified using classical criteria (Lewis et al. 1977, Ready et al. 1980), this catalogue follows the phylogenetic classification proposed by Galati (1995). The article is presented in accordance with earlier lists of other insect groups (Zompro and Domenico 2005, Mello and Ziegler 2012, Reboleira et al. 2012). Image database and digitalization of information has been undertaken and the latter is available at Species Link Database (http://splink.cria.org.br).

The taxon and respective available data are listed below in alphabetical order of genus using the following format:

***Binomial name*** Author, publication date.

Journal or Technical Bulletin, volume (number): pages.

**Typology:** Specimens’ number and gender. Information for all specimens was a direct copy of the labels with no modification. Labels are indicated by quotation marks (“ ”), with each line in the label separated by a double slash (//), and handwriting information in labels indicated by italics.

**Type-locality:** locality or municipality (geographical coordinates), State or department, country.

**Original name:** *as in the original description*.

**Additional note:** only Brazilian institutions were assessed. Characteristics of each type series were highlighted giving the number of slides and number of specimens mounted on them, mentioning if the specimens were dissected or remounted or the presence of a label on the slide.

### Unusual abbreviations are listed below:

Coll. collector

Coll. site collection site

Det. Determinator

Dept. department

Met. Method of capture

sp. n. new species

rem. remounted

SH. Shannon trap

Sp. species name

## Results

The following annotated catalogue to each type species deposited in the ColFleb IEC (splink.cria.org.br/) is given below.

### List of type material

***Nyssomyia richardwardi* (Ready & Fraiha, 1981)**

Revista Brasileira de Biologia 41(4): 705–712.

**Paratypes:** 1 female “Wellcome // Parasitology Unit // Slide n°*44.17* // Sp *Lu. richardwardi* // Det: *Paratype* // Coll. Site *Km 43* // *Altamira- Itaituba*// *Pará- Brasil* // Met. *Igarapé* // *Isca humana* // Date 21/8/1971” [ColFleb IEC 149]; 1 female “Wellcome // Parasitology Unit // Slide *44.18* // Sp *Lu. richardwardi* // Det: *Paratype* // Coll. Site *Km 43* // *Altamira- Itaituba*// *Pará- Brasil* // Met. *Igarapé* // *Isca humana* // Date 21/8/1971” [ColFleb IEC 150].

**Type-locality:** km 43 of the Transamazonian Highway, between Itaituba (04°16'33"S, 55°59'02"W) and Altamira (03°11'41"S, 52°12'33"W) municipalities, State of Pará, Brazil.

**Original name:** *Lutzomyia richardwardi*.

**Additional note:** two slides.

***Nyssomyia shawi* (Fraiha, Ward & Ready, 1981)**

Revista Brasileira de Biologia 41(4): 699–703.

**Paratypes:** 4 females “Wellcome // Parasitology Unit // Slide n°*43.‘N’* // Sp *Lu. shawi* // Det: *Paratype* // Coll. Site // *Serra dos Carajás* // *Marabá, Pará* // Met. *Isca humana* // Date *20/9/1974*” [‘*N*’= slide n°43.15, 43.25, 43.28, 43.29] [ColFleb IEC 159– 162]; 8 females “Wellcome // Parasitology Unit // Slide *43‘N*’ // Sp *Lu. shawi* // Det: *Paratype* // Coll. Site // *Serra dos Carajás* // *Marabá- Pará* // Met. *Isca humana* // Date *22/9/1974*” [‘*N*’= slide n°43.09, 43.14, 43.16, 43.17, 43.23, 43.24, 43.26, 43.27] [ColFleb IEC 163–170]; 6 females “Wellcome // Parasitology Unit // Slide n°*43‘N*’ // Sp *Lu. shawi* // Det: *Paratype* // Coll. Site // *Serra dos Carajás* // *Marabá- Pará* // Met. *Isca humana* // Date *23/9/1974*” [‘*N*’= slide n°43.02, 43.03, 43.04, 43.05, 43.07, 43.08] [ColFleb IEC178– 183]; 7 females “Wellcome // Parasitology Unit // Slide *43. ‘N*’ // Sp *Lu. shawi* // Det: *Paratype* // Coll. Site // *Serra dos Carajás* // *Marabá- Pará* // Met. *Isca humana* // Date *9/1974*” [‘*N*’= slide n°43.01, 43.06, 43.12, 43.13, 43.18, 43.21, 43.22] [ColFleb IEC 171–177]; 9 males “Wellcome // Parasitology Unit // Slide n°*43.‘N’* // Sp *Lu. shawi* // Det: *Paratype* // (*Serra dos carajás* // Coll. Site *Marabá*- // *Pará- Fevereiro 1975*) // Met. *Criado em lab.* // Date *Abril- Junho 1975* // *R.D. Ward col.*” [‘*N*’= slide n°43.44, 43.45, 43.48, 43.49, 43.50, 43.51, 43.53, 43.61, 43.62] [ColFleb IEC 184–192].

**Type-locality:** Serra dos Carajás (06°06'29"S, 50°18'36"W), Marabá municipality, state of the Pará, Brazil.

**Original name:** *Lutzomyia shawi*.

**Additional note:** 34 slides.

***Nyssomyia umbratilis* (Ward & Fraiha, 1977)**

Journal of Medical Entomology 14(3): 313–317.

**Holotype:** female “Wellcome // Parasitology Unit // *Lu. umbratilis* // Slide n°*Holótipo* // Sp *260.65* // Det.” [ColFleb IEC 145].

**Paratype:** 1 female “Wellcome // Parasitology Unit // Coll. Site *Area 2*’ // *Lu umbratilis* // *Jari* // Met. *Tree trunks* // Date *19/7/76* // *Lu. umbratilis*” [ColFleb IEC 146].

**Type-locality:** Monte Dourado (00°53'23"S, 52°36'08"W), locality, Almeirim municipality, state of Pará, Brazil.

**Original name:** *Lutzomyia (Nyssomyia) umbratilis*.

**Additional note:** The two females mounted on the same glass slide. The locality of Jari actually refers to Monte Dourado, in Almeirim municipality, State of Pará.

***Nyssomyia yuilli yuilli* (Young & Porter, 1972)**

Journal of Medical Entomology 9(6): 524–526.

**Paratypes:** 1 male “*Colombia #179* // *Antioquia dept.*, // *24Km south and* // *21 Km west of* // *Zaragoza.* // *in light trap* // *3 may 1970* // *coll: C.M. Porter* // *D L503-D1202-1* // *Lutzomyia* // *yuilli* // *Young* // *Porter* // *Paratype* // *Det: D.G. Young*” [ColFleb IEC 157]; 1 female “*Colombia* // *Antioquia dept.*, // *24Km south and* // *21 Km west of* // *Zaragoza.* // *in light trap* // *3 may 1970* // *coll: C.M. Porter* // *D L503-D1202-1* // *Lutzomyia* // *yuilli* // *Young* // *Porter* // *Paratype* // *Det: D.G. Young*” [ColFleb IEC 156].

**Type-locality:** Zaragoza (07°29'23"N, 74°52'03"W), Antioquia Department, Colombia.

**Original name:** *Lutzomyia yuilli*.

**Additional note:** two slides.

***Pintomyia gruta* (Ryan, 1986)**

Flebótomos do Estado do Pará, Brasil (Diptera, Psychodidae, Phlebotominae). Documento Técnico. n°1, Instituto Evandro Chagas, Belém, Pará, 154 p.

**Paratypes:** 1 male “Wellcome ♂ // Parasitology Unit // Slide n°*Paratipo* // Sp *Lu. gruta* // Det: *Lee Ryan* // Coll. Site *Gruta* // *Carajás NI* // Met. *CDC* // Date: *28/3/1985*” [ColFleb IEC 139]; 1 male “Wellcome ♂ // Parasitology Unit // *Paratipo* // Slide n°// Sp *Lu. gruta* // Det: *Lee Ryan* // Coll. Site *Gruta* // *Carajás NI* // Met. *CDC* // Date: *28.3.85*” [ColFleb IEC 140]; 1 male “Wellcome ♂ // Parasitology Unit // *Paratipo* // Slide n°// Sp *Lu. gruta* // Det: *Lee Ryan* // Coll. Site *Gruta* // *Carajás NI* // Met. *CDC* // Date: *28.3.85*” [ColFleb IEC 142]; 1 female “Wellcome ♀ // Parasitology Unit // *Paratipo* // Slide n°// Sp *Lu. gruta* // Det: *Lee Ryan* // Coll. Site *Carajás* // *NI Gruta no. 2* // Met. *CDC* // Date: *14/3/86*” [ColFleb IEC 141]; 1 female “*diss* Wellcome ♀ // Parasitology Unit //Slide n°*Paratipo* // Sp *Lu. gruta* // Det: *Lee Ryan* // Coll. Site *Carajás* // *NI Gruta* // Met. *CDC* // Date: *10/2/1985*” [ColFleb IEC 138].

**Type-locality:** Serra dos Carajás (06°06'29"S, 50°18'36"W), Marabá municipality, state of the Pará, Brazil.

**Original name:** *Lutzomyia gruta*.

**Additional note:** five slides. The term “diss” in the beginning of the label ColFleb IEC 183 refers to dissected specimens.

***Psychodopygus lainsoni* Fraiha & Ward, 1974**

Bulletin of Entomological Research 64: 209–212.

**Syntypes:** 1 male “*Psychodopygus* // *lainsoni sp. n.* // *Síntipo* ♂- *Km 46* // *Altamira- Itaituba* // *Rod. Transamazônica* // *Pará- Brasil Km 46 Altamira- Itaituba Rod. Transamazônica, Pará, Brasil* // *Criado em laboratório* // *Ps. lainsoni* ♂” [ColFleb IEC 131]; 1 male “*Psychodopygus* // *lainsoni sp. n.* // *Síntipo* ♂- *Km 46* // *Altamira- Itaituba* // *Rod. Transamazônica* // *Pará- Brasil Km 46 Altamira- Itaituba Rod. Transamazônica, Estado do Pará* // *Criado em laboratório*” [ColFleb IEC 132].

**Type-locality:** km 46 of the Transamazonian Highway, between Itaituba (04°16'33"S, 55°59'02"W) and Altamira (03°11'41"S, 52°12'33"W) municipalities, state of the Pará, Brazil.

**Original name:** *Psychodopygus lainsoni*

**Additional note:** one slide with two specimens. No label. Data written directly on the slide with white brush.

***Psychodopygus leonidasdeanei* Fraiha, Ryan, Ward, Lainson & Shaw, 1986**

Memórias do Instituto Oswaldo Cruz 81(3): 333–339.

**Holotype:** male “Wellcome // Parasitology Unit // Slide n°? // Sp *Lu. leonidasdeanei* // Det: *Lee Ryan* // Coll. Site *Santarém* // *Serra do BEC* // Met: *SH (2)* // *20/7/1984* // *18- 19hs*” [ColFleb IEC 121].

**Paratypes:** 1 female “Wellcome // Parasitology Unit // Slide n°// Sp *Lu leonidas* // Det *deanei* // *Transamazônica* // Coll. Site *Km 112* // *Itaituba- Altamira* // Met: *isca humana* // *18:19hs* // Date *30/11/71*” [ColFleb IEC 130]; 42 females “Wellcome // Parasitology Unit // *Lu leonidasdeanei* // Slide n°// Sp // Det // Coll. Site *Km 112* // *Itaituba- Altamira* // Met: *Isca humana* // Date *3-4-75* // *Fraiha & Ward col*.” [ColFleb IEC 1–6, 27, 31–33, 37, 39-42, 48, 50, 52, 53, 55, 56, 60–62, 64, 65, 67, 69–72, 77, 83, 97, 101, 102, 104, 106, 107, 117, 118, 120]; 1 female “Wellcome // Parasitology Unit // *Lu leonidasdeanei* // Slide n°// Sp // Det // *Vicinal 11/13 Km 46* // Coll. Site *Altamira* // *Itaituba- Lote 39* // Met: *Isca humana* // Date *18.12.73* // *Fraiha col.*” [ColFleb IEC 11]; 2 females “Wellcome // Parasitology Unit // Slide n°*sp. grp*. // Sp *Lu. leonidasdeanei* // Det *Lee ryan* // Coll. Site Santarém // Serra do BEC // Met SH (2) 18-19hs // *date* 10.7.1984 [ColFleb IEC 122 (allotype), 123].

**Type-locality:** Santarém (02°24'52"S, 54°42'36"W) municipality, State of the Pará, Brazil.

**Original name:** *Psychodopygus leonidasdeanei*

**Additional note:** To the male holotype the sentence “met: SH (2)” refers to Shannon trap with two collectors.

***Psychodopygus llanosmartinsi* Fraiha & Ward, 1980**

Revista da Fundação de Serviços de Saúde Pública 25(1): 10–14.

**Paratypes:** 1 female “Loc *Pilcopata* // *Dep. Cuzco* // Estado // *Peru* // Cap por *J.E.* // *Silva* // Em 21/6/73 // Lam n°*46.293* // NE n°1905/73 // Esp *L. amazo* // *nensis* // Det. *A.V. Martins* // Em *Set. 1973.* [ColFleb IEC 158]; 1 female. “Loc *Pilcopata* // *Dep. Cuzco* // Estado // *Peru* // Cap por *J.E.* // *Silva* // Em 21/6/73 // Lam n°*46.297* // NE n°1905/73 // Esp *L. amazo* // *nensis* // Det. *A.V. Martin* // Em *Set. 1973*” [ColFleb IEC 159].

**Type-locality:** Pilcopata (02°30'00"S, 78°55'00"W), Cuzco Department, Peru.

**Original name:** *Psychodopygus llanosmartinsi*.

**Additional note:** two slides. The original label refers to the specimens as *Lutzomyia amazonensis*.

***Psychodopygus wellcomei* Fraiha, Shaw & Lainson, 1971**

Memórias do Instituto Oswaldo Cruz 69(3): 489–500.

**Syntypes:** 1 female “*Psychodopygus* // *wellcomei* // *Fraiha*, *Shaw & lain-* // *son, 1971, Sintipo* ♀ // *Serra Norte, Area 2* // *Serra dos Carajás* // *Marabá, Pará, BR* // *Isca Humana* // *Área de cerrado* // *na borda da mata* // *23. Nov. 1968* [ColFleb IEC 133]; 1 male “*P. wellcomei* // *Fraiha*, *Shaw* // *& Lainson, 1971* // (*capt. em armad.* // *Disney c/ cobaio* // *Psychodopygus* // *wellcomei* // *Sintipo* ♂ // *Serra Norte- A1* // *Serra dos Carajás* // *Marabá, Pará, BR* // *23-24/XI/1968*” [ColFleb IEC 134].

**Type-locality:** Serra dos Carajás (06°06'29"S, 50°18'36"W), Marabá municipality, State of the Pará, Brazil.

**Original name:** *Psychodopygus wellcomei*.

**Additional note:** two slides.

***Trichophoromyia adelsonsouzai* Santos, Silva, Barata, Andrade & Galati, 2014**

Memórias do Instituto Oswaldo Cruz 109: 2014 (ahead of print).

**Holotype:** male “Universidade de São Paulo // Faculdade de Saúde Pública // Epidemiologia // BRASIL – Pará // Vitória do Xingu entre Altamira e // Anapu (km 27 da R. Transamzônica) // Armadilhas CDC e Shannon // Cols. Santos TV & Silva FMM, // 04-15/II/2012 a 10-26/VVI/2012 // *Trichophoromyia adelsonsouzai* // Santos, Silva, Barata, Andrade & // Galati 2013/ Holótipo ♂ [Colfleb IEC 184].

**Paratypes:** 13 males “Universidade de São Paulo // Faculdade de Saúde Pública // Epidemiologia // BRASIL – Pará // Vitória do Xingu entre Altamira e // Anapu (km 27 da R. Transamzônica) // Armadilhas CDC e Shannon // Cols. Santos TV & Silva FMM, // 04-15/II/2012 a 10-26/VVI/2012 // *Trichophoromyia adelsonsouzai* // Santos, Silva, Barata, Andrade & // Galati 2013 [ColFleb IEC 185–194]; 5 females “Universidade de São Paulo // Faculdade de Saúde Pública // Epidemiologia // BRASIL – Pará // Vitória do Xingu entre Altamira e // Anapu (km 23 da R. Transamzônica) // Armadilhas CDC e Shannon // Cols. Santos TV & Silva FMM, // 04-15/II/2012 a 10-26/VVI/2012 // *Trichophoromyia adelsonsouzai* // Santos, Silva, Barata, Andrade & // Galati 2013 [ColFleb IEC 195–199].

**Type-locality:** km 47 of the Transamazonian Highway, between Altamira (03°11'41"S, 52°12'33"W) and Anapu (03°28'21"S, 51°11'55"W) municipalities, State of the Pará, Brazil.

**Original name:** *Trichophoromyia adelsonsouzai*.

**Additional note:** 19 slides.

***Thichophoromyia brachipyga* (Mangabeira, 1942)**

Memórias do Instituto Oswaldo Cruz 37(2): 111–218.

**Paratype:** 1 male “170 // Inst. Osw. Cruz // N^o^. *Paratypo* // *F. brachipygus* // *Mangabeira*, *1942* // *Piratuba, M. Aba-* // *eté (Pará) em bura-* // *co de tatu- VIII- 940* // *Mang. coll*.” [ColFleb IEC 148].

**Type-locality:** Abaetetuba (01°43'04"S, 48°52'58"W) municipality, State of the Pará, Brazil.

**Original name:** *Flebotomus brachipygus*.

**Additional note:** one slide. The municipality of Abaeté actually refers to Abaetetuba.

***Thrichophoromyia readyi* (Ryan, 1986)**

Flebótomos do Estado do Pará, Brasil (Diptera, Psychodidae, Phlebotominae), Documento técnico n°1, Instituto Evandro Chagas, Belém, Pará, 154 p.

**Holotype:** 1 male “*holotype* Wellcome // Parasitology Unit // *remounted 1.11.85* // Slide n°*L. Ryan* // Sp *Lu. ready* // Det: *R. D. Ward* // *Transamazônica* // Coll. Site *Km 25* // *Itaituba- Altamira* // Met. *toca tatu* // Date 29/11/71 // *coll R.D.W.*” [ColFleb IEC 142].

**Paratypes:** 1 female “Wellcome // Parasitology Unit // *allotype* // Slide n°*R.D. Ward collect* // Sp *Lu. ready* // Det: *Lee Ryan* // *Transamazon* // Coll. Site *Km 25* // *Itaituba- Altamira* // Met. *CDC* // Date 2.12.71” [ColFleb IEC 143 (allotype)]; 1 male “Wellcome // Parasitology Unit // *R.D.Ward* // Slide n°*340.61* // Sp *Lu. ready* // Det: *Lee Ryan* // *Transamazon* // Coll. Site *Km 25* // *Itaituba- Altamira* // Met. *Armadillo burrow* // Date *29.11.1971* // *rem. 10.2.1986*” [ColFleb IEC 145].

**Type-locality:** km 25 of the Transamazonian Highway, between Itaituba (04°16'33"S, 55°59'02"W) and Altamira municipalities (03°11'41"S, 52°12'33"W), State of the Pará, Brazil.

**Original name:** *Lutzomyia readyi*.

**Additional note:** three slides. Paratype [ColFleb IEC 145] was remounted on 10/II/1986 and the holotype on 1/XI/1985.

## Conclusion

Until now, there are 12 sand fly species included as type specimens deposited in the COLFleb IEC. The type material of *Evandromyia carmelinoi*, including male holotype and the female described as allotype (= paratype), is lost. The other two species mentioned in the original description as deposited in the “Instituto Evandro Chagas”, *Bichromomyia olmeca nociva* and *Psychodopygus yucumensis* were not found in the collection. According to original description the species described by Mangabeira during 1938 and 1942 were deposited in the “Instituto Oswaldo Cruz” and those by Damasceno and collaborators and between 1944 and 1950 in the Natural History Museum of the United States (Smithsonian Museum, Washington D.C.).

The ColFleb IEC is the only Institute in Brazil that holds type–species of *Pintomyia gruta*, *Psychodopygus leonidasdeanei* and *Thrichophoromyia readyi* (AJA, personal observation). The male holotype and 2 male and three female paratypes of *Psychodopygus lainsoni* are deposited at the “Coleção Padrão” of the “Departamento de Epidemiologia” of the “Faculdade de Saúde Pública” of the “Universidade de São Paulo” (FSP–USP). Three other type series of sand flies can be found in the same Institution: two female paratypes of *Nyssomyia umbratilis* and a male and a female “topotypes” of *Nyssomyia yuilli yuilli*. In the “Coleção de Flebotomíneos” of the “Centro de Pesquisas René Rachou” (FIOCRUZ-COLFLEB) another slide of a *Psychodopygus llanosmartinsi* male, described as “plesiotype/paratype”, was found in addition to two paratype males (n°44.38–44.39) and six paratype females (n°44.20–44.25) of *Nyssomyia richardwardi*, and four paratype males (n°43.52, 43.54, 43.55, 43.56) and six paratype female (n°43.30–43.35) of *Nyssomyia shawi*. With regards to *Psychodopygus wellcomei*, one slide of a syntype female is deposited in the FIOCRUZ-COLFLEB, and one syntype male and two syntype females of the same species are found in the entomological collection named “Costa Lima” in the Instituto Oswaldo Cruz (CEIOC). According to Fraiha and Ryan (1986) more than 20 valid species were described by Dr. Octavio Mangabeira Filho. Unfortunately, few specimens described by him have been found in Brazilian institutions (Andrey Andrade, personal communication). Of these, just one male paratype of *Trichophoromyia brachipyga* was located in the ColFleb IEC and another male paratype is deposited in the “John Lane Collection” of the FSP-USP. Finally, paratypes of *Thrichophoromyia adelsonsouzai* have been distributed to different collections in Brazil (Santos et al. 2014).

Depository institutions and natural history collections are very important to know and document biodiversity around the world. Unfortunately, these holdings of biodiversity information are often not easy to survey and it is common to observe that some type species are not deposited according to the original description. This may be due to the fact that the author does not always send them to the intended institution, or the specimens are later donated or sold to other institutions, or sadly they were destroyed or lost. Annotated catalogues are the first step to organize these invaluable collections and they may help in updating the deposited material. The authors hope that similar studies can be made by other institutions which have relevant collections, particularly those that hold type–species of Brazilian species.
